# Editorial: Progress in Episodic Memory Research

**DOI:** 10.3389/fnbeh.2016.00061

**Published:** 2016-03-30

**Authors:** Ekrem Dere, Armin Zlomuzica, Angelica Staniloiu, Hans J. Markowitsch

**Affiliations:** ^1^Teaching and Research Unit Life Sciences, University Pierre and Marie CurieParis, France; ^2^Max Planck Institute of Experimental MedicineGöttingen, Germany; ^3^Mental Health Research and Treatment Center, Ruhr-University BochumBochum, Germany; ^4^Department of Physiological Psychology, University of BielefeldBielefeld, Germany; ^5^Sunnybrook Health Science CenterToronto, ON, Canada; ^6^Department of Psychiatry, University of TorontoToronto, ON, Canada

**Keywords:** episodic memory, episodic-like memory, episodic future thinking, mental time travel, animal feed

Episodic memory refers to the ability to mentally time travel into the past and to remember personal experiences in terms of what happened and where and when it happened and to be autonoetically aware of it (Tulving, 2002). Humans (and perhaps also animals) are able to remember the specific perceptions, emotions and thoughts they had during a particular experience. This highly sophisticated and unique memory system is extremely sensitive to cerebral aging, neurodegenerative and neuropsychiatric diseases.

Episodic memory, as the sum of our experiences, is the supporting pillar of our identity and reminds us about our preferences and aversions, our strengths and weaknesses and helps us to anticipate how we will think, feel and behave in the future. Therefore, episodic memories are extremely useful for solving problems in the present and to plan for the future (Breeden et al., 2016).

The field of episodic memory research is a continuously expanding and fascinating area that unites a broad spectrum of scientists who represent a variety of research disciplines including neuroscience, behavioral genetics, medicine, neuropharmacology, psychology and philosophy. Nevertheless, important questions still remain to be addressed.

This research topic on the Progress in Episodic Memory Research covers past, current and future directions in research dedicated to the neurobiology, neuropathology, development, measurement and rehabilitation of episodic memory. A total of 171 high-rank international scientists have contributed to a compilation of 43 articles.

Among these 43 articles the reader will find (already well cited) original research (Alston et al.; Bai et al.; Barbosa et al.; Baumann et al.; Chen et al.; Coman et al.; Dalton et al.; Denkova et al.; Denkova et al.; Guillery-Girard et al.; Habermas and Diel; Henke et al.; Kanatsou et al.; Kinugawa et al.; Meyer et al.; Mulder et al.; Rauchs et al.; Reber et al.; Risius et al.; Shafer and Dolcos; Sperduti et al.; Staniloiu et al.; Todd et al.; Xiu et al.), opinion (Szpunar et al.) and review articles (Brod et al.; Friedman; Griffin and Hallock; Irish and Piguet; Mizumori; Muller; Murray and Kensinger; Pause et al.; Pergola and Suchan; Schnider; Souchay et al.; Werner et al.; Zlomuzica et al.), as well as hypothesis and theory papers (Dalla Barba and La Corte; Klein; Urbanowitsch et al.; Vandekerckhove et al.; Yassa and Reagh) from both human and animal research disciplines.

With this *Frontiers eBook*, we (the editors) aimed to cover the full diversity of methods and theoretical approaches to unravel this veritable “marvel of nature” (Tulving, 2002) that we call episodic memory.

## Author contributions

All authors listed, have made substantial, direct and intellectual contribution to the work, and approved it for publication.

### Conflict of interest statement

The authors declare that the research was conducted in the absence of any commercial or financial relationships that could be construed as a potential conflict of interest.
